# Health-Related Behaviors of Pregnant Women Residing in Urban and Rural Areas in Poland

**DOI:** 10.3390/ijerph17124395

**Published:** 2020-06-18

**Authors:** Maria Szubert, Malwina Ilowiecka, Jacek Wilczynski, Przemyslaw Bilinski, Cezary Wojtyla

**Affiliations:** 1 Clinic of Surgical and Oncologic Gynecology, 1st Department of Gynecology and Obstetrics, Medical University of Lodz, M. Pirogow Teaching Hospital, Wilenska 37 St., 94-029 Lodz, Poland; maja.szubert@gmail.com (M.S.); malilo1@o2.pl (M.I.); jrwil@post.pl (J.W.); 2 Club 35, Polish Society of Gynecologists and Obstetricians, Cybernetyki 7F/87 St., 02-677 Warsaw, Poland; 3 Faculty of Health Sciences, State University of Applied Sciences, Kaszubska 16 St., 62-800 Kalisz, Poland; bildom@gmail.com; 4 International Prevention Research Institute—Collaborating Centre, State University of Applied Sciences, Kaszubska 16 St., 62-800 Kalisz, Poland; 5 Department of Oncologic Gynecology and Obstetrics, Centre of Postgraduate Medical Education, Czerniakowska 231 St., 00-416 Warsaw, Poland

**Keywords:** health behaviors, pregnancy, rural area, urban area

## Abstract

The aim of this study was to evaluate the knowledge regarding a healthy lifestyle and prophylaxis during pregnancy among women from rural and urban areas and how this changed within a 5-year period. Analyses of the population of pregnant women in Poland were made using a questionnaire survey. The survey was conducted in the years 2010–2012 and 2017. Questionnaires from 6128 pregnant women were collected. The statistical analyses were conducted using IBM SPSS. The examined population was comprised of 41% women from rural areas and 59% women from urban areas. Alcohol consumption was lower among women from rural areas than among urban inhabitants in 2010–2012; in 2017 a trend of even lower consumption was observed. Folic acid supplementation was more broadly developed in the urban population; however, in 2017, higher percentage rates of both populations admitted taking folates before pregnancy. More women in urban than in rural areas performed physical activity during pregnancy, but the differences decreased in 2017. Knowledge of a healthy lifestyle and prophylaxis during pregnancy increased regardless of place of residence; however, the most evident change could be observed among women from rural areas.

## 1. Introduction

Contemporary medicine offers access to tools and options that may not only reduce the prevalence of certain diseases or modify disease-related complications but also extend lifespan and improve its quality. According to the report published by M. Lalonde in 1974, our lifestyle determines our health by as much as 50%, whereas the influence of healthcare itself is limited only to 10% [1]. Almost 45 years have passed since this theory was presented, and although many new statements have been made since that time, Lalonde’s health field concept clearly reflects the significance of health prevention. An advanced development in medicine may not be an argument for diminishing the impact that lifestyle exerts on overall health. Reasonable behaviors aimed at the protection and improvement of health are particularly important in pregnant women since each mother’s lifestyle has a great impact on the course of pregnancy and fetal development [2]. Therefore, both worldwide and in Poland, research studies have been conducted regularly with the aim of evaluating knowledge on pro-health factors and behavior among future mothers [3,4]. The identification of risk factors and assessment of health awareness, particularly in populations of pregnant women, creates the option to implement specific actions and projects aimed at ensuring proper care or at filling in knowledge gaps related to the aforementioned issues. Thus, we undertook this study in two specific subgroups: rural and urban populations of pregnant women. It is worthwhile to mention that, from 2009, recommendations regarding nearly all aspects of pregnancy were released in Poland which were intended to be implemented irrespective of the place of residence of the future mother. Such studies are also important because they show whether such actions bring certain measurable benefits, such as a reduced number of diseases and complications, which in turn may be translated into lower financial outlays for secondary prevention. Based on the published analyses on health awareness among pregnant women, it may be concluded that the results are influenced by similar factors to those among non-pregnant women, such as age, place of residence, level of education and marital and socioeconomic status. These factors indicate that there is a positive correlation between a more advanced age of a pregnant woman, a higher education level, a higher socioeconomic status and a greater care for health [5,6]. The noticeable differences in the abovementioned observations resulted in the focusing of educational activities on populations in which the awareness of health prevention importance is still too low. One of the population groups in East-Central Europe whose economic situation has greatly improved in the recent years is the inhabitants of rural regions. The Regulation of the Minister of Health of 16 August 2018 on the organizational standard of perinatal care obliged medical professionals in Poland to take the same pro-health actions among pregnant patients regardless of their place of residence or financial status [7].

The aim of this study was to analyze health awareness, expressed both as knowledge and pro-health behaviors, in the populations of pregnant women living in urban and rural areas. Based on these data, we attempted to assess changes in health awareness in the two populations within a five-year observation period; i.e., between the years 2010–2012 and 2017. The working hypothesis assumed that, despite place of residence, women should have a comparable health awareness, which should increase in the studied timeframe.

## 2. Materials and Methods

The study was conducted by the Chief Sanitary Inspectorate and the Institute of Rural Health in the years 2010–2012 and in 2017 in all Polish public hospitals and involved a group of women and their children. The analysis was made based on a questionnaire developed within Pol-PrAMS program (Polish Pregnancy-Related Assessment Monitoring System) using the model of the PRAMS (Pregnancy Risk Assessment Monitoring System) applied in the United States. All women gave their informed consent for inclusion before they participated in the study. The Bioethics Committee of the Institute of Rural Health in Lublin approved the study (permission no. 03/2011).

The participants in the Pol-PrAMS study were 12.066 pregnant women, including 8625 subjects in the years 2010–2012 and 3441 in 2017. Women hospitalized in public hospitals in Poland for delivery were asked to fill in the paper version of the questionnaire, which was divided into two parts. The first one was filled in by the patients and referred to their personal data, pro-health behaviors and awareness of a healthy lifestyle during pregnancy and the course of pregnancy, whereas the second part was filled in by healthcare professionals based on medical records of the patients and their children. The Pregnancy Physical Activity Questionnaire (PPAQ) was used to estimate the physical activity of pregnant women in both groups. The use of PPAQ allowed the presentation of the physical activity of pregnant women as their energy expenditure, measured in Metabolic Equivalents of Task (METs). Physical activity is presented in MET-h/week units. The Mann–Whitney–Wilcoxon test was used to compare groups in relation to physical activity. The detailed methodology of the Pol-PrAMS study and the use of PPAQ has been presented in separate studies [8,9].

The demographic population structure in 2010–2012 was significantly different to that studied in 2017 in terms of age and region of residence. A compromise method of correction was adopted by randomly withdrawing those cases that were overrepresented [10]. The population from the 2010–2012 study was referenced to the population structure from 2017 in terms of age, place of residence (city/village) and region of residence (16 provinces). This data constituted a trivariate table, covering 3331 women subjects from 2017 divided into 3 × 3 × 16 = 144 subgroups. The number of subjects in each subgroup was expressed as a percentage of the total. An analogous table was prepared for the population studied in 2010–2012. Based on the coefficients from the 2017 table, the expected counts in each subgroup for 2010–2012 were calculated, from which the overcounts and undercounts could be derived. The population size was reduced so that there were no undercounts present in the table; i.e., only overcounts were present. This was achieved by reducing the total size of the interviewed population from the 2010–2012 study to 2797 cases. Current and newly expected counts were provided for each subgroup. The required number of cases was then randomly selected using the “SAMPLE n from m” procedure from the SPSS database program, containing the data for each subgroup of the 2010–2012 study. This procedure randomly selects an m-element sample of cases from an n-element population [11]. In total, 144 of these procedures were separately performed for the subgroups. The adjusted study population of 2797 cases did not differ significantly from the 2017 study population in terms of the unified variable structure. Obtained data were compared between the years of the survey in women residing urban and rural areas. The statistical analysis of the collected data was made based on a chi-squared test using IBM SPSS software version 25 (IBM Corp., Armonk, NY, USA).

Due to the fact that some women did not answer some questions, the sample size in the description of the results is smaller than the actual number of women included for the study; thus, the percentage value is the so-called valid percentage, calculated for the number of answers and not the number of completed questionnaires.

## 3. Results

### 3.1. Characteristics of the Study Population

The largest part of the study group, both in the years 2010–2012 and in 2017, included women aged between 26 and 30 years; the smallest one comprised women aged over 35 years. Among the surveyed participants, women living in urban areas accounted for around 59%, whereas those from rural regions accounted for around 41% of the study group. The highest percentage in both groups was represented by women with a normal body weight before pregnancy—i.e., their Body Mass Index (BMI) was between 18.5–24.9 kg/m^2^—and they constituted about 67%. The average weight gain of pregnant women in the years 2010–2012 was 14.4 kg, while in 2017 it was 14.6 kg. Characteristics of the study population are presented in Table 1.

### 3.2. Alcohol and Stimulant Use During Pregnancy

Cigarette smoking by pregnant women living in both urban and rural areas decreased over the years (Table 2). In the group of women living in urban areas, about 16% of women quit smoking because of pregnancy, both in 2010–2012 and in 2017. This percentage increased from 14.0% to 15.9% in the group of women living in rural areas.

Women living in rural areas consumed alcohol less frequently than those living in cities, both before and during pregnancy (Table 2). In 2010–2012 and 2017, 36.8% and 33.1% of women living in rural areas consumed alcohol before pregnancy, respectively, while the percentages among women living in the city were 45.1% and 40.8%, respectively. Alcohol consumption during pregnancy in rural areas was 15.4% in 2010–2012 and 3.5% in 2017, and for city inhabitants, these values were 18.7% and 6.0%, respectively.

In the study population, 0.1–0.4% of women used legal highs during pregnancy; the percentage rate remained at a very low level both in urban and rural areas (Table 2).

### 3.3. Folic Acid Supplementation

A majority of the study participants from both rural and city regions began folic acid supplementation during pregnancy—in the years 2010–2012, 67.4% of the women living in rural areas and 62% of those living in urban areas supplemented their diet with folic acid, and in 2017, the values were 66.9% and 62.8%, respectively (Table 2). However, a significantly higher percentage of women living in urban areas started to supplement folic acid before pregnancy and continued supplementation during pregnancy compared to women from rural regions. The dose of folic acid taken was not studied.

### 3.4. Types of Beverages

Considering the types of beverages consumed, the women most frequently chose water. As many as 84% of urban population studied in the years 2010–2012 and 85.6% of individuals analyzed in 2017 declared drinking water every day, whereas the women living in rural areas declared drinking higher amounts of sweetened beverages and tea. In the years 2010–2012, the percentage rate was 23% for sweetened beverages and 74.1% for tea, and in 2017, the values were 10.1% for sweetened beverages and 64.1% for tea. Coffee consumption decreased among women living in rural and urban areas during the analyzed years (Table 2).

### 3.5. Physical Activity

The median for the total value of physical activity in the years 2010–2012 was 200.3 MET-h/week in women living in urban areas and 188.1 MET-h/week in those living in rural regions. In 2017, the respective figures were 195.6 MET-h/week in urban areas and 178.4 in rural areas. Over the analyzed years, the only differences observed in the rural population were related to sedentary physical activity. The energy expenditure related to household activity among women living in urban areas increased significantly in the analyzed period of time; however, it decreased significantly in the case of occupational physical activity. Women living in urban areas more often engaged in occupational physical activity in pregnancy as well as sport activity, expressed in MET-h/week, compared to rural inhabitants. Physical activity of analyzed groups of women is presented in Table 3.

### 3.6. Body Mass Index

The distribution of BMI among women living in urban and rural areas was comparable in 2010–2012, whereas in 2017, in the rural population, both before pregnancy and in the prenatal period, the percentage rate of overweight women as well as those with class I (BMI 30–34.9 kg/m^2^), class II (BMI 35–39.9 kg/m^2^) and class III obesity (BMI ≥ 40 kg/m^2^) was higher than among those living in urban areas. Comparing the groups of women from both analyzed periods of time, there were no statistically significant differences observed in relation to BMI before delivery in the rural and urban population (Table 4).

## 4. Discussion

To the best of our knowledge, this is the first study on changes in health awareness in the population of pregnant women residing in urban and rural areas in Poland. Understanding these changes is important for healthcare providers to improve counselling during pregnancy and improve maternal and newborn health outcomes. Rural areas in Poland occupy more than 93% of the country’s area; this high share automatically becomes a source of causes and effects for differences both at the regional and local level [12]. Despite this fact, health awareness in the rural population, both expressed as better knowledge and healthier behaviors declared by surveyed patients, has been increasing dynamically. These changes in the rural population of pregnant women are probably related to improvements in road infrastructure and access to the Internet, but these aspects were not studied in our survey [13]. The increasing health awareness might be also influenced by an increase in gross domestic product (about 230% in 2016 in comparison to 2002) in the rural population [14].

The analysis of the rural population clearly demonstrates a decrease in the number of births and professional activity and an increase in expenditure on health and education (in the years 1999–2004). An additional aspect is the constantly improving access to out-patient health care in rural areas [15]. Additionally, the nature of migration from rural to urban areas has been changing; increasingly often, people living in rural regions give up permanent migration and decide to choose temporary migration [14].

Currently, the healthcare offered to pregnant women focuses on the promotion of the so-called healthy lifestyle already in the preconception period. In pregnancy, the first medical appointment should be arranged by the 10th week of gestation, and it should include counselling with regard to diet, folic acid intake, non-use of stimulants and smoking cessation [7,16,17]. It is particularly important that a healthcare professional and a pregnant woman should cooperate in order to prepare a schedule of prenatal care that should be modified as required by the patient’s health condition and needs. Thus, it is possible to achieve the best possible results and avoid mistakes that could lead to complications and failures.

A permanent problem faced by the Polish society, despite numerous social campaigns, is increasing alcohol consumption [18]. The problem of consuming both hard and weak liquors is also present for pregnant women. This is confirmed by the fact that the National Health Plan for the Years 2016–2020 includes a point on the dissemination of knowledge regarding the harmful effects resulting from alcohol consumption by pregnant women [19]. Moreover, although this aspect remains ignored, it is crucial for women to realize the importance of abstaining from drinking alcohol even in the preconception period; in particular, it should be stressed that the organogenesis stage lasts from the second until the end of the eighth week, which is particularly important given that a large number of women find it difficult to define the exact day of conception [20].

A survey performed in 2012 showed that urban inhabitants consume alcohol more frequently than the rural population [21]; this is probably the reason why this trend is also observed in the group of pregnant women included in our study. A positive phenomenon, however, is the fact that the consumption of alcohol among women living in rural as well as in urban areas has decreased over the analyzed years.

Studies conducted in Poland show that smoking only depends on place of residence to a small extent. In 2016, it was proved that women living in small towns with up to 20,000 inhabitants were addicted to smoking less frequently. The highest percentage rate of female smokers was reported in cities with 100,000–500,000 inhabitants [22].

Our study did not find any statistically significant differences regarding changes in smoking habits in pregnancy in the analyzed period of time, either in rural and urban areas. In addition, the percentage of smoking women living in rural and urban areas remained at a comparable level. In literature sources, it can increasingly often be seen that a negative factor in smoking cessation is not only a low socioeconomic status but also the fact that women ignore the harmful effects of the addiction if their previous child was born healthy. Factors that limit smoking cessation also include a partner who is a smoker, as well as a belief that the addiction may be a stress-reducing activity [23,24]. Due to the large scale and topicality of the problem, not only does an attending doctor play a great role in increasing the awareness of women, but also midwives, childbirth education institutions and individuals are important advocates in a pregnant woman’s immediate environment. Importantly, a woman’s attention should also be focused on reducing passive smoking as a negative factor for the health of the offspring [25].

Some studies show that women are more often successful at resisting such addictions as cigarettes, alcohol or caffeinated drinks than, for example, at increasing amounts of vegetables or fruit in their diet [26]. Our study partly confirmed these data. About 14–16% of women quit smoking during pregnancy in the analyzed period, while a significant part of them stopped drinking alcohol in connection with pregnancy, especially among women participating in the study in 2017. Apart from personal attitude and motivation, healthy lifestyle is also influenced by individual (such as financial and time-related) and infrastructural options. Although a size of a place of residence determines physical activity to a small extent only, the greatest percentage rate of active individuals was observed among people living in cities with more than 500,000 inhabitants, which may result from the better sport facilities available [27]. In our study, the mean total energy expenditure of women living in cities increased over the analyzed years, in contrast to women living in rural areas. In addition, this expenditure among urban residents was higher than among women living in rural areas.

A similar dependency exists regarding the consumption of sweetened beverages in the rural and urban populations. This may be partly explained by the unfavorable economic situation of rural areas; however, the phenomena that may influence this situation have not yet been fully investigated [28]. Nevertheless, it needs to be emphasized that the opinions given by the women participating in our survey show that the consumption of sweetened beverages among the rural population has decreased significantly in the last few years, and the figures are at present only slightly higher than those for the women representing the urban population.

An alarming occurrence is an increasing number of rural inhabitants who are overweight or suffer from class I, II and III obesity, which reflects a general worldwide trend [29,30]. Studies show that obese individuals are not able to classify their body mass or do not realize that it is abnormal. Moreover, it has been found that partners of overweight or obese persons deal with the same problem, which may be an additional factor causing difficulties in maintaining normal bodyweight before and during pregnancy [31]. Consultation with an attending doctor, proper preparation and the discussion of common objectives, including the maximum weight gain during pregnancy, should be one of the main issues within a preconception care scheme offered to women who plan to become pregnant. Obesity also generates disturbances in levels of microelements crucial for fetal development; e.g., folic acid [32].

The influence of folic acid on the development of the fetal nervous system was observed as early as in the 1960s by the British physician Bryan Hibbard, while, in 1991, the Medical Research Council proved in its study that daily intake of 4 mg of folic acid may prevent defects of the fetal neural tube in women who had previously given birth to a child with such an anomaly [33]. More recent research studies have demonstrated that women of reproductive age planning pregnancy should commence folic acid supplementation already at 12 weeks before the planned pregnancy [34,35]. According to our data, a majority of women begin to take folic acid after they have their pregnancy confirmed; this is particularly evident in women representing the rural population. Only every third women surveyed was aware of the importance of folic acid supplementation before conception and the recommended dose; this may suggest that only 30% of pregnancies were planned, but this aspect was not assessed in this study. In contrast, among female transplant recipients, only every third pregnancy is unplanned [36]. Family planning is therefore of high importance when discussing health awareness among pregnant women. The targeted promotion of folic acid supplement use should be conducted periodically by gynecologists and primary care physicians during annual medical screenings [37].

Our study suggests that, despite the universal access to information for both rural and urban inhabitants and development of prenatal care, there is still a need for education about proper nutrition and other health-related issues regardless of place of residence. These suggestions are also supported by Ługowska et al. and Dereń et al. [38,39] and in other populations [40]. Progress in antenatal care has been made worldwide, which is also supported by our results [41]. Some studies indicate that not only should recommendations be implemented but also “active clinical mentoring” (including such activities as organizing and training a national team of mentors including senior midwives, obstetricians and pediatricians) should be used to improve health awareness even before pregnancy [42]. The meta-analysis by Miteniece E et al. indicated other issues that should be improved, with the geographical distance to healthcare institutions being one of the most important [43].

## 5. Conclusions

In the last decade, there has been a considerable change in pro-health behaviors in the population of women living in rural areas and a slight change among those living in cities. Although the results of studies seem to be satisfactory, there are still aspects that should be changed in both of the groups. At the same time, as a result of globalization, other problems may arise in Polish rural and urban populations, and they could occur paradoxically in both of the groups, posing new challenges to doctors and other medical professionals providing care to pregnant women.

## Figures and Tables

**Table 1:** Characteristics of the study population.

Variable	2010–2012		2017		*p*
	N	%	N	%	
Age (years)					0.91
Under 25	532	19.0	627	19.0	
26–30	965	34.6	1139	34.6	
31–35	925	33.1	1073	32.6	
Over 35	371	13.3	457	13.8	
Place of residence (inhabitants)					0.99
City ≥ 100,000	676	24.2	804	24.1	
City < 100,000	967	34.6	1154	34.7	
Rural area	1154	41.2	1373	41.2	
BMI before pregnancy (kg/m^2^)					0.2
<18.5	230	8.4	256	7.9	
18.5–24.9	1838	67.6	2147	66.6	
25.0–29.9	471	17.3	581	18.0	
30.0–34.9	143	5.3	176	5.5	
35.0–39.9	25	0.9	53	1.6	
≥40	13	0.5	13	0.4	
**Parameter**	**Mean**	**S.D.**	**Median**		***p***
Weight before pregnancy (kg)					0.29
2010–2012	63.5	12.2	61.0		
2017	63.9	12.5	62.0		
Weight gain in pregnancy (kg)					0.4
2010–2012	14.4	5.6	13.0		
2017	14.6	6.2	14.0		

**Table 2:** Alcohol consumption, stimulant use, folic acid supplementation and consumption of various types of drinks in connection with pregnancy in urban and rural areas in Poland, in the period of 2010–2012 and 2017.

	Urban Area			Rural Area		
	2010–2012	2017	*p*	2010–2012	2017	*p*
	% (N)	% (N)		% (N)	% (N)	
Smoking			0.1			0.65
Yes	7.1 (77)	5.4 (104)		6.4 (49)	6.0 (80)	
No	76.6 (829)	78.6 (1508)		79.6 (611)	78.1 (1042)	
Quit in pregnancy	16.3 (176)	16.0 (306)		14.0 (107)	15.9 (212)	
Alcohol						
Before pregnancy	45.1 (675)	40.8 (798)	0.01	36.8 (370)	33.1 (454)	0.06
During pregnancy	18.7 (276)	6.0 (118)	0.00	15.4 (152)	3.5 (48)	0.00
Legal highs			0.43			0.62
Yes	0.4 (4)	0.2 (4)		0.1 (1)	0.2 (3)	
No	99.6 (1075)	99.8 (1867)		99.9 (764)	99.8 (1304)	
Folic acid supplementation						
Before and during pregnancy	29.5 (445)	32.3 (598)	0.08	21.0 (211)	27.1 (332)	0.00
During pregnancy	62.0 (937)	62.8 (1164)	0.63	67.4 (675)	66.9 (818)	0.81
Without supplementation	8.5 (129)	4.9 (91)	0.00	11.6 (116)	6.0 (73)	0.00
Water			0.2			0.38
Never	2.4 (38)	1.6 (31)		3.2 (34)	2.3 (30)	
Sometimes	13.6 (214)	12.8 (241)		18.5 (197)	17.8 (227)	
Every day	84.0 (1319)	85.6 (1612)		78.2 (831)	79.9 (1020)	
Sweetened beverages			0.00			0.00
Never	30.1 (440)	38.7 (673)		28.0 (268)	32.5 (378)	
Sometimes	51.3 (750)	51.8 (900)		49.0 (469)	57.4 (667)	
Everyday	18.6 (272)	9.4 (164)		23.0 (220)	10.1 (117)	
Tea			0.00			0.00
Never	4.8 (75)	4.4 (81)		3.8 (40)	3.1 (40)	
Sometimes	27.5 (425)	37.4 (695)		22.1 (232)	32.8 (418)	
Everyday	67.7 (1048)	58.3 (1083)		74.1 (777)	64.1 (817)	
Cofee			0.00			0.00
Never	40.6 (600)	41.1 (730)		45.0 (434)	42.7 (510)	
Sometimes	38.0 (561)	43.6 (775)		34.0 (328)	42.2 (503)	
Everyday	21.4 (317)	15.4 (273)		21.0 (203)	15.1 (180)	

**Table 3:** Physical activity of women during pregnancy living in rural and urban areas in Poland in the years 2010–2012 and 2017.

	N	Mean	S.D	Median	First Quartile	Third Quartile	*p*
Urban area							
Total physical activity							0.97
2010–2012	1059	215.3	116.3	200.3	140.2	265.6	
2017	1897	217.3	117.5	195.6	139.8	270.8	
Household activity							0.00
2010–2012	1048	102.8	77.3	80.5	49.6	136.9	
2017	1880	112.1	81.7	92.1	52.3	150.3	
Occupational activity							0.00
2010–2012	595	38.4	62.0	14	0.0	67.2	
2017	1094	33.2	59.0	0.0	0.0	56.0	
Sport/execrice activity							0.88
2010–2012	1029	7.6	8.8	4.8	2.4	9.6	
2017	1858	8.0	9.8	4.8	2.4	9.6	
Sedentary activity							0.00
2010–2012	1048	69.9	41.5	65.9	39.6	97.8	
2017	1872	60.4	38.0	54.1	30.6	87.0	
Moderate-intensity activity							0.04
2010–2012	1056	53.7	64.1	34.4	9.6	71.0	
2017	1890	58.6	70.4	36.0	13.1	78.6	
Vigorous-intensity activity							0.00
2010–2012	928	0.9	3.1	0.0	0.0	0.0	
2017	1747	1.25	3.9	0.0	0.0	0.0	
Rural area							
Total physical activity							0.39
2010–2012	762	206.2	118.2	188.1	126.5	268.3	
2017	1313	204.2	119.9	178.4	125.6	251.5	
Household activity							0.2
2010–2012	753	110.9	85.5	92.1	50.9	145.8	
2017	1302	113.2	81.6	93.6	54.4	150.3	
Occupational activity							0.16
2010–2012	429	31.0	49.5	0.0	0.0	56.0	
2017	710	30.4	60.5	0.0	0.0	52.9	
Sport/execrice activity							0.22
2010–2012	732	5.9	7.1	3.6	1.7	8.1	
2017	1268	6.2	9.2	3.4	0.8	8.0	
Sedentary activity							0.02
2010–2012	742	58.1	40.3	48.2	28.7	84.7	
2017	1292	52.6	35.3	47.3	25.6	75.7	
Moderate-intensity activity							0.69
2010–2012	754	56.6	66.0	35.3	10.1	77.8	
2017	1306	55.5	69.4	32.6	10.5	74.6	
Vigorous-intensity activity							0.09
2010–2012	647	0.64	2.73	0.0	0.0	0.0	
2017	1175	0.94	3.5	0.0	0.0	0.0	

**Table 4:** Body Mass Index (BMI) of women living in rural and urban areas in Poland, before pregnancy and before labor, in the years 2010–2012 and 2017.

	Urban Area			Rural Area		
	2010–2012	2017	*p*	2010–2012	2017	*p*
	% (N)	% (N)		% (N)	% (N)	
BMI before pregnancy (kg/m^2^)			0.8			0.12
<18.5	8.7 (139)	8.5 (159)		8.2 (91)	7.3 (97)	
18.5–24.9	68.2 (1094)	68.8 (1306)		66.7 (744)	63.3 (841)	
25.0–29.9	16.8 (270)	16.4 (311)		18.0 (201)	20.3 (270)	
30.0–34.9	5.0 (80)	4.8 (92)		5.7 (63)	6.3 (84)	
35.0–39.9	0.8 (13)	1.2 (23)		1.1 (12)	2.3 (30)	
≥40.0	0.6 (9)	0.4 (7)		0.4 (4)	0.5 (6)	
BMI before labor (kg/m^2^)			0.39			0.05
<18.5	0.1 (1)	0.1 (2)		0.1 (1)	0.1 (1)	
18.5–24.9	25.1 (402)	24.3 (458)		21.7 (241)	19.3 (254)	
25.0–29.9	46.8 (751)	45.7 (860)		49.2 (547)	45.9 (605)	
30.0–34.9	21.6 (347)	21.5 (404)		21.6 (240)	24.9 (328)	
35.0–39.9	5.4 (86)	6.9 (130)		6.0 (67)	7.4 (98)	
≥40.0	1.1 (17)	1.5 (28)		1.4 (16)	2.5 (33)	
